# Exploring the relationship between *IGHMBP2* gene mutations and spinal muscular atrophy with respiratory distress type 1 and Charcot-Marie-Tooth disease type 2S: a systematic review

**DOI:** 10.3389/fnins.2023.1252075

**Published:** 2023-11-17

**Authors:** Yuan Tian, Jinfang Xing, Ying Shi, Enwu Yuan

**Affiliations:** ^1^Department of Clinical Laboratory, The Third Affiliated Hospital of Zhengzhou University, Zhengzhou, China; ^2^Screening Center, The Third Affiliated Hospital of Zhengzhou University, Zhengzhou, China

**Keywords:** *IGHMBP2* gene, spinal muscular atrophy with respiratory distress type 1, Charcot-Marie-Tooth disease, mutations, clinical diagnosis

## Abstract

**Background:**

*IGHMBP2* is a crucial gene for the development and maintenance of the nervous system, especially in the survival of motor neurons. Mutations in this gene have been associated with spinal muscular atrophy with respiratory distress type 1 (SMARD1) and Charcot-Marie-Tooth disease type 2S (CMT2S).

**Methods:**

We conducted a systematic literature search using the PubMed database to identify studies published up to April 1st, 2023, that investigated the association between *IGHMBP2* mutations and SMARD1 or CMT2S. We compared the non-truncating mutations and truncating mutations of the *IGHMBP2* gene and selected high-frequency mutations of the *IGHMBP2* gene.

**Results:**

We identified 52 articles that investigated the association between *IGHMBP2* mutations and SMARD1/CMT2S. We found 6 hotspot mutations of the *IGHMBP2* gene. The truncating mutations in trans were all associated with SMARD1.

**Conclusion:**

This study provides evidence that the complete LOF mechanism of the *IGHMBP2* gene defect may be an important cause of SMARD1.

## Introduction

*IGHMBP2* is a gene located on chromosome 11 that encodes the immunoglobulin mu-binding protein 2, also known as SMN-interacting protein 1 (SIP1) (Surrey et al., 2018). This protein plays a crucial role in the development and maintenance of the nervous system, particularly in the survival of motor neurons (Rzepnikowska et al., 2022). The protein contains various domains, including an ATPase domain, a RNA helicase domain, and a zinc finger domain, which are involved in RNA processing and transport and the regulation of gene expression (Rzepnikowska and Kochanski, 2021). The helicase core of the IGHMBP2 protein is comprised of four distinct regions, including two RecA-like domains (Domains 1A and 2A) with two subdomains (1B and 1C) inserted into Domain 1A (Lim et al., 2012). The regions of Domains 1A (residues 159–270 and 347–440) and 2A (residues 441–648) that are highly conserved are critical for the proper function of the IGHMBP2 protein (Saladini et al., 2020). Mutations in these regions have been associated with spinal muscular atrophy with respiratory distress type 1 (SMARD1) (Pekuz et al., 2022).

Spinal muscular atrophy with respiratory distress type 1 (SMARD1) is a rare genetic disorder characterized by progressive muscle weakness and respiratory distress in early infancy, which is caused by mutations in the *IGHMBP2* gene (Perego et al., 2020). The symptoms of SMARD1 typically appear in the first few months of life and include muscle weakness, particularly in the diaphragm and other muscles involved in breathing, as well as decreased reflexes, difficulty swallowing, and impaired movement (Saeed et al., 2021). The disorder can lead to respiratory failure and death in severe cases (Bodle et al., 2021).

In recent years, increasing reports have suggested that mutations in the *IGHMBP2* gene may cause CMT2S, in addition to SMARD1 (Lei et al., 2022). CMT2S is a subtype of Charcot-Marie-Tooth disease (CMT) which is a hereditary neuropathy that affects the peripheral nervous system (Chandrasekharan et al., 2022). It is characterized by muscle weakness and wasting in the distal limbs, sensory loss, and reduced or absent tendon reflexes (Xie et al., 2021). Compared to SMARD1, CMT2S has a milder phenotype and does not typically present with respiratory distress or spinal motor neuron loss. Literature has indicated that mutations in the 5′ region and the last exon of the *IGHMBP2* gene are predominantly associated with CMT2, including nonsense mutations, frameshift mutations, missense mutations, and compound heterozygous mutations (Saladini et al., 2020).

Due to that different mutations in the *IGHMBP2* gene can lead to either SMARD1 or CMT2S, the identification of the specific mutations in *IGHMBP2* that lead to SMARD1 or CMT2S is important for clinical diagnosis, treatment, and genetic counseling. According to these, our study aims to explore the relationship between different types of *IGHMBP2* gene mutations and the two distinct disease types they cause by retrieving relevant literature from the PubMed database, screening and analyzing the retrieved articles. Our goal is to identify the specific regions of the *IGHMBP2* gene where mutations occur and their correlation with these two different diseases. The ultimate objective of this study is to provide more precise genetic counseling for clinical diagnosis and treatment.

## Methods

### Literature search using PUBMED

**Figure 1:**
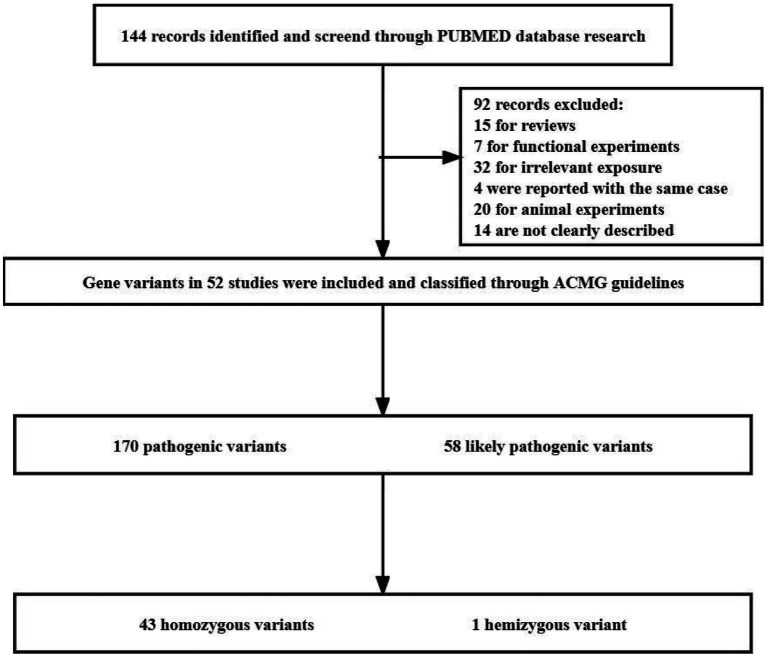
Flow diagram of included studies.

We conducted a systematic literature search using the PubMed database to identify studies published up to April 1st, 2023 that investigated the association between *IGHMBP2* gene mutations and Charcot-Marie-Tooth disease, spinal muscular atrophy with respiratory distress, distal hereditary motor neuronopathy, and autosomal recessive distal spinal muscular atrophy. We used the search terms “IGHMBP2” and “Charcot-Marie-Tooth disease” or “spinal muscular atrophy with respiratory distress” or “distal hereditary motor neuronopathy” or “autosomal recessive distal spinal muscular atrophy.” The final search equation was defined using the Boolean connectors “AND” and “OR,” as follows: “IGHMBP2” AND {“Charcot-Marie-Tooth disease” OR “spinal muscular atrophy with respiratory distress” OR “distal hereditary motor neuronopathy” OR “autosomal recessive distal spinal muscular atrophy”}. We excluded non-original works not related to human subjects, such as reviews, functional experiments on animals or cells, the same cases, not clearly described studies and original articles that had no information relevant to the purpose of this study. After identifying the relevant articles, we classified the pathogenicity of the *IGHMBP2* gene mutations involved in the articles according to the American College of Medical Genetics and Genomics (ACMG) guidelines (Richards et al., 2015). Pathogenicity was classified as “Pathogenic (P),” “Likely pathogenic (LP),” “Uncertain significance (VUS),” “Benign (B),” or “Likely benign (LB).” Finally, these reported P/LP homozygous variants were selected for distribution analysis in the gene structure and protein functional regions. The literature search and analysis process are shown in Figure 1.

### Comparative analysis of *IGHMBP2* gene mutations from literature search grouped by SMARD1 and CMT2S

We calculated the percentage of *IGHMBP2* gene mutations identified through the literature search that were associated with SMARD1 and CMT2S, respectively. We compared the non-truncating mutations located in RecA-like domains (Domains 1A and 2A) of the *IGHMBP2* gene between the SMARD1 and CMT2S groups to determine if there were any differences. We also compared the homozygous non-truncating mutations in the *IGHMBP2* gene between the SMARD1 and CMT2S groups. In addition, we compared the truncating mutations located in the last exon of the *IGHMBP2* gene between the SMARD1 and CMT2S groups and assessed the homozygous truncating mutations in each group.

### Distribution analysis of gene structure regions and protein functional domains for homozygous variants in the *IGHMBP2* gene

We selected 43 homozygous and 1 hemizygous (with heterozygous deletion of exons 6–13) *IGHMBP2* gene variants classified as P/LP according to ACMG guidelines, and classified them into truncating and non-truncating variants. We analyzed the distribution of these variants in the gene structure and protein functional regions, and created separate lollipop plots for each type of variant. We performed SWISS-MODEL homology modeling and used SPDBV_4.10 software to analyze the 3D protein structure of the missense variants in these homozygous variants.

### Data summary and analysis of high-frequency IGHMBP2 gene mutations

We selected *IGHMBP2* gene mutations that appeared in 5 or more probands in the literature search and considered them to be high-frequency mutations. We analyzed the number of times these mutations were associated with SMARD1 and CMT2S, respectively, as well as their trans position. We then identified any patterns or relationships between these mutations and the two diseases.

### Statistical analysis

In this study, statistical comparisons of data were performed using the χ^2^ test (*p* values less than 0.05 were considered statistically significant), and all data analyses were conducted using SPSS 22.0. Graphs were plotted using IBS 2.0 and Graphpad prism 9.

## Results

### Percentage of *IGHMBP2* gene mutations identified through the literature search that were associated with SMARD1 and CMT2S

**Figure 2:**
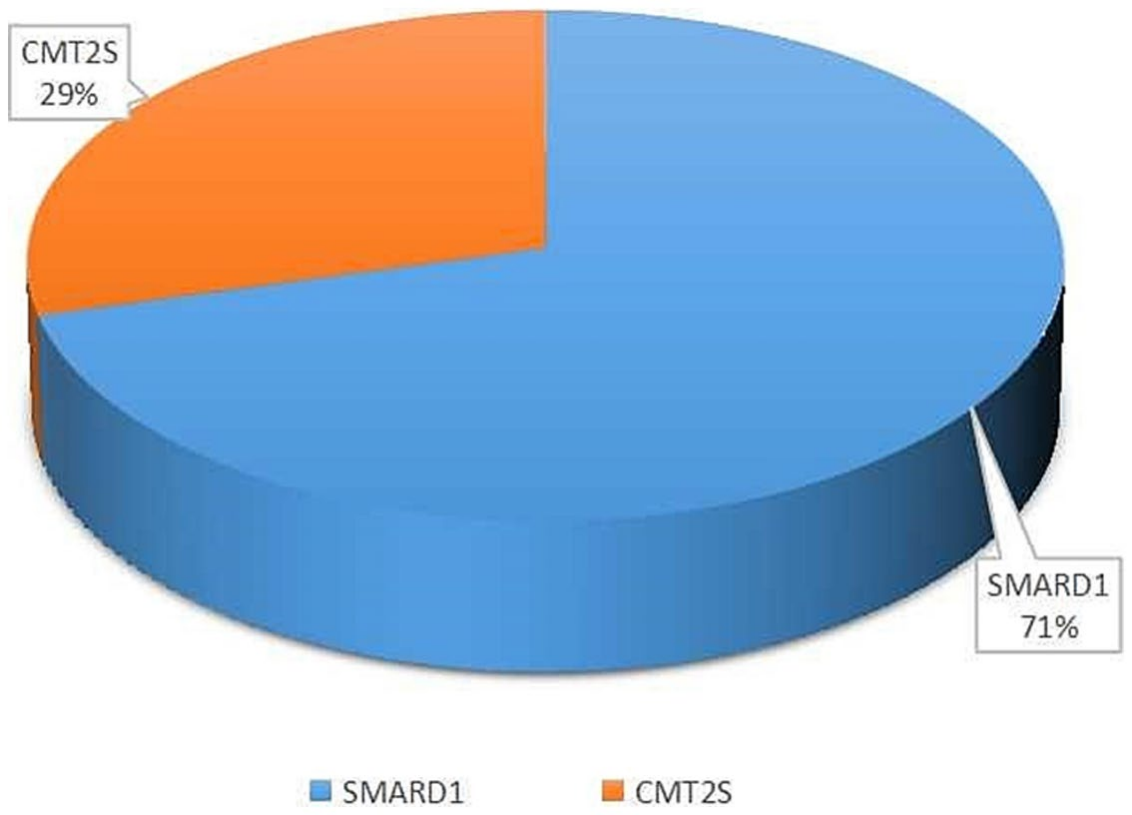
The percentage of SMARD1 and CMT2S within the entirety of literature data.

Among the 52 literature articles screened (Supplementary Table S1) (Robison et al., 1998; Grohmann et al., 2001, 2003; Guenther et al., 2004, 2007; Maystadt et al., 2004; Giannini et al., 2006; Wong et al., 2006; Joseph et al., 2009; AlSaman and Tomoum, 2010; Baughn et al., 2011; Pierson et al., 2011; Chalancon et al., 2012; Eckart et al., 2012; Majid et al., 2012; Messina et al., 2012; Gitiaux et al., 2013; Blaschek et al., 2014; Cottenie et al., 2014; Jedrzejowska et al., 2014; Lin et al., 2014; Litvinenko et al., 2014; Hamilton et al., 2015; Han et al., 2015; Wagner et al., 2015; Lingappa et al., 2016; Luan et al., 2016; Pedurupillay et al., 2016; San et al., 2016; Dohrn et al., 2017; Liu et al., 2017; Yuan et al., 2017; Zhang et al., 2017; Habibi et al., 2018; Kulshrestha et al., 2018; Tomaselli et al., 2018; Wu et al., 2018; Cassini et al., 2019; Kim et al., 2019; Yasui et al., 2019; Cortese et al., 2020; Tsang et al., 2020; Bodle et al., 2021; Felice et al., 2021; Saeed et al., 2021; Xie et al., 2021; Berti et al., 2022; Chandrasekharan et al., 2022; Megarbane et al., 2022; Pekuz et al., 2022; Taiana et al., 2022; Xiao et al., 2022), SMARD1 accounted for 71% and CMT2S accounted for 29% of the mutations associated with the study’s purpose (Figure 2).

### Comparison of the non-truncating mutations located in RecA-like domains (domains 1A and 2A) of The *IGHMBP2* gene between the SMARD1 and CMT2S

Among SMARD1-associated non-truncating mutations (missenese and inframe) in the *IGHMBP2* gene, 90.4% (66/73) were located in RecA-like domains (Domains 1A and 2A), while in CMT2S-associated non-truncating mutations, 71.1% (27/38) were located in these domains. Statistical analysis showed a significant difference between the two diseases in terms of the RecA-like domains (Domains 1A and 2A) mutations (χ^2^ = 6.893, *p* = 0.009) (Table 1).

**Table 1:** Comparison of non-truncating *IGHMBP2* gene variant positions and types between SMARD1 and CMT2S.

Disease	Whether is in recA-like domains (domains 1A and 2A)		Variant type(s)	
	Yes	No	Homozygous	Compound heterozygous
SMARD1	66	7	19	54
CMT2S	27	11	4	34
*χ* ^2^	6.893		3.655	
*p*	0.009		0.056	

### Comparison of the truncating mutations located in the last exon in the *IGHMBP2* gene between the SMARD1 and CMT2S

Among SMARD1-associated truncating mutations (nonsense, frameshift and splice) in the *IGHMBP2* gene, 1.2% (1/82) were located in the last exon, while in CMT2S-associated truncating mutations, 15.4% (4/26) were located in the last exon. Statistical analysis showed a significant difference between the two diseases in terms of mutations located in the last exon (χ^2^ = 8.971, *p* = 0.012) (Table 2).

**Table 2:** Comparison of truncating *IGHMBP2* gene variant positions and types between SMARD1 and CMT2S.

Disease	Whether is in the last exon		Variant type(s)	
	Yes	No	Homozygous	Compound heterozygous
SMARD1	1	81	20	62
CMT2S	4	22	8	18
*χ* ^2^	8.971		0.418	
*p*	0.012		0.518	

### Comparison of the homozygous mutations in the *IGHMBP2* gene between the SMARD1 and CMT2S

In SMARD1-associated mutations in the *IGHMBP2* gene, homozygous non-truncating mutations accounted for 26.0% (19/73) of all mutations, while in CMT2S-associated non-truncating mutations, homozygous mutations accounted for 10.5% (4/38) (Table 1). Statistical analysis showed no significant difference between the two diseases in terms of homozygous mutations (χ^2^ = 3.655, *p* = 0.056) (Table 1). Besides, homozygous truncating mutations accounted for 24.4% (20/82) of all mutations, while in CMT2S-associated truncating mutations, homozygous mutations accounted for 30.8% (8/26) (Table 2). Statistical analysis showed no significant difference between the two diseases in terms of homozygous mutations (χ^2^ = 0.418, *p* = 0.518) (Table 2).

**Figure 3:**
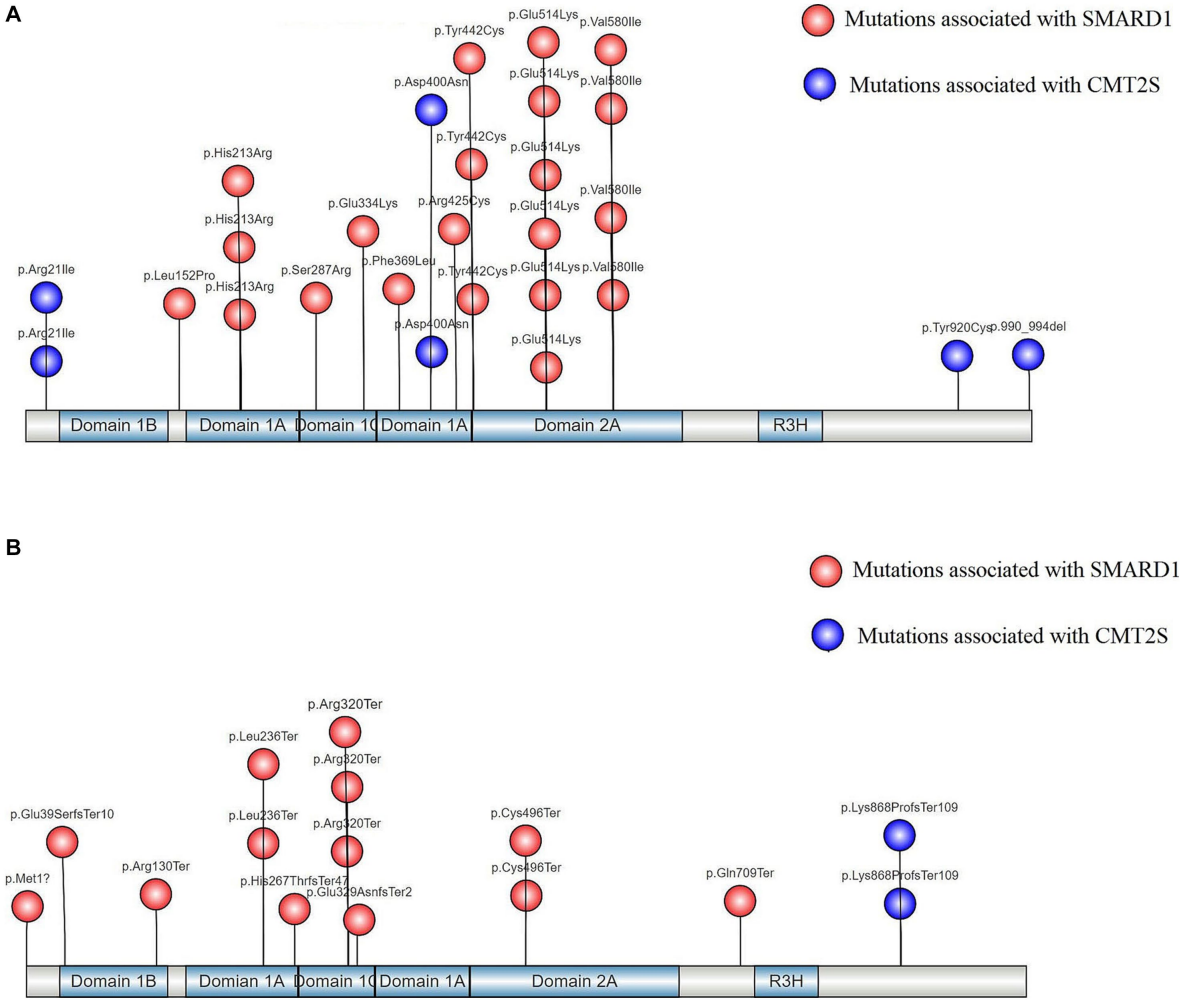
**(A)** Distribution of gene structure and protein functional regions of non-truncating mutations in *IGHMBP2* gene after literature screening; **(B)** Gene structure and protein functional domain distribution of *IGHMBP2* gene with truncating mutations identified through literature screening.

After analyzing 43 homozygous variants and 1 hemizygous variant (Supplementary Table S2) of the *IGHMBP2* gene, we found that non-truncating variants located at the 5′ and 3′ ends of the gene are associated with CMT2S, while non-truncating variants associated with SMARD1 are mostly located within the two RecA-like domains (Domains 1A and 2A) with two subdomains (1B and 1C) (Figure 3A). The only exception is the p.Asp400Asn variant, which is located in Domain 1A but has been reported to be associated with CMT2S (Figure 3A). Among these homozygous truncating mutations in the *IGHMBP2* gene, only the p.Lys868ProfsTer109 mutation is associated with CMT2S, while all other truncating homozygous mutations have been reported to be associated with SMARD1 (Figure 3B).

**Figure 4:**
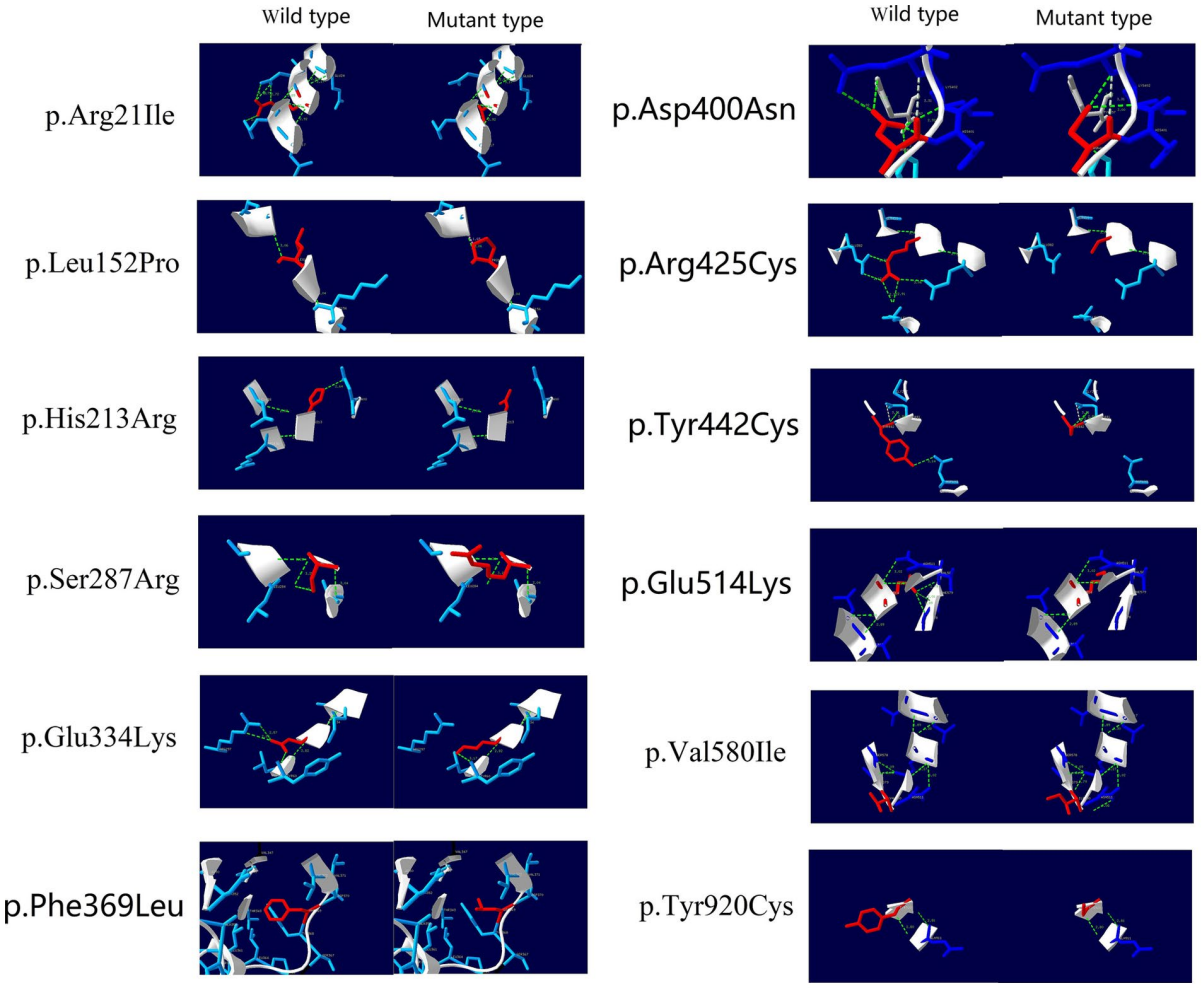
3D Structure prediction of missense variants in *IGHMBP2* Gene after literature screening.

After conducting protein 3D structure analysis on the missense variants among these homozygous variants, we found that, except for the hemizygous variant p.Phe369Leu, all the homozygous variants showed changes in hydrogen bonds relative to the wild-type protein, resulting in decreased protein stability and changes in the protein 3D structure (Figure 4).

### Data summary and analysis of high-frequency IGHMBP2 gene mutations

**Figure 5:**
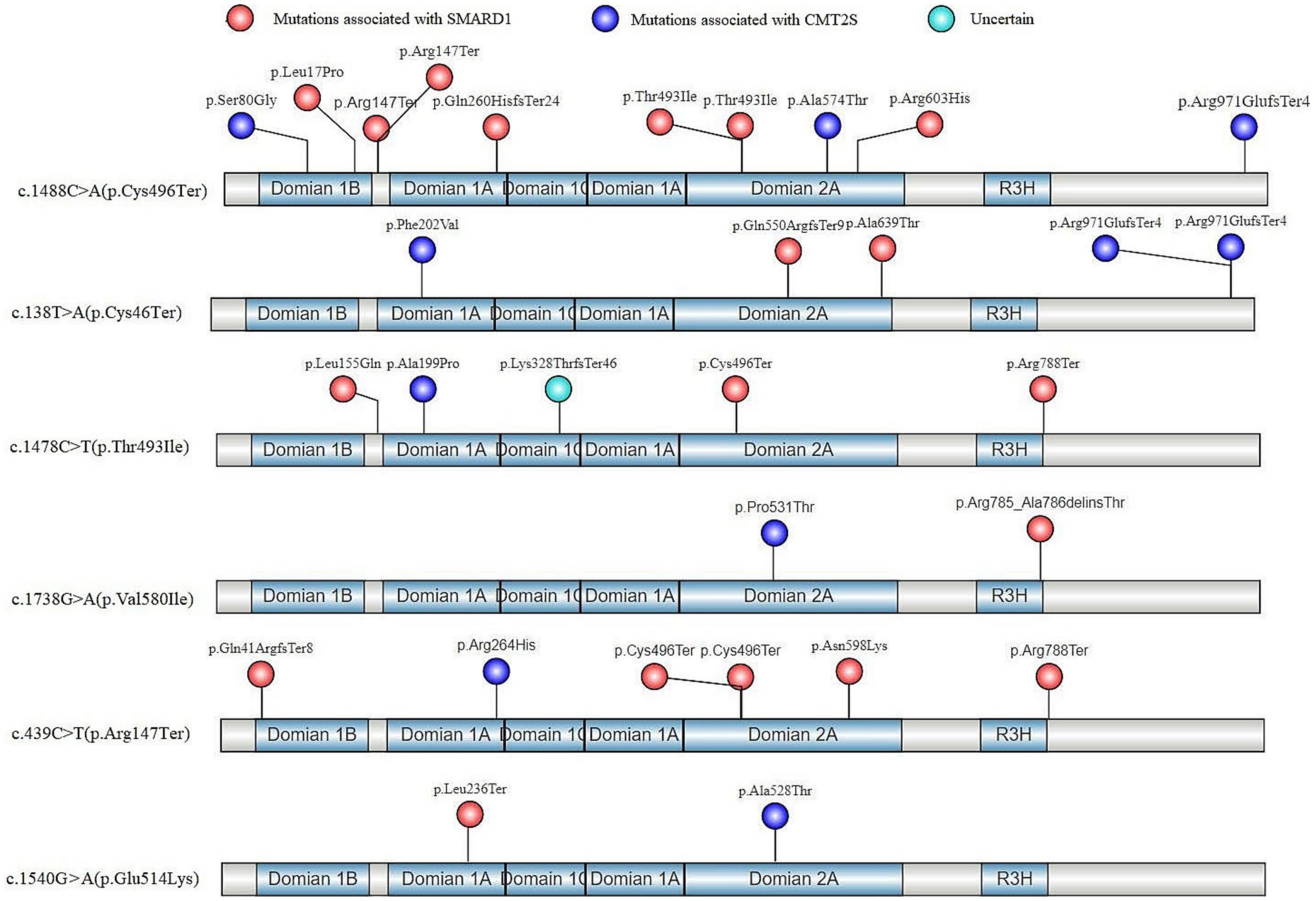
In-trans mutations of hotspot variants in the gene structure and protein functional regions of *IGHMBP2* gene after literature screening.

Based on the identified high-frequency mutations in the *IGHMBP2* gene, 6 hotspot mutations were determined: c.1488C > A (p.Cys496Ter) (Cottenie et al., 2014; San et al., 2016; Dohrn et al., 2017; Cortese et al., 2020), c.138 T > A (p.Cys46Ter) (Grohmann et al., 2003; Taiana et al., 2022), c.1478C > T (p.Thr493Ile) (Eckart et al., 2012; Hamilton et al., 2015; Pedurupillay et al., 2016), c.1738G > A (p.Val580Ile) (Wong et al., 2006), c.439C > T (p. Arg147Ter) (Jedrzejowska et al., 2014), and c.1540G > A (p.Glu514Lys) (Grohmann et al., 2001), among which c.1488C > A (p.Cys496Ter) had the highest incidence rate (13/42) (Supplementary Table S3). All of these 6 mutations exhibited phenotypic variability, with SMARD1 having a higher incidence rate (30/42) overall (Supplementary Table S3). Our analysis of the in-trans variants of these 6 hotspot mutations in terms of gene structure and protein functional domains revealed that, except for the p.Arg971GlufsTer4 mutation which is associated with CMT2S, all other truncating variants were associated with SMARD1 (Figure 5). Non-truncating mutations in these in-trans variants did not show a clear regional distribution pattern (Figure 5).

## Discussion

*IGHMBP2* gene mutations are associated with two diseases, SMARD1 (Rzepnikowska et al., 2022) and CMT2S (Martin et al., 2022), which exhibit significant differences in phenotype. Therefore, understanding the detection rate of these two diseases is important. In this study, a literature search has revealed that SMARD1 accounted for 71% of all IGHMBP2-related diseases detected, making it the most significant disease type caused by mutations in this gene.

The human *IGHMBP2* gene consists of 15 exons and encodes immunoglobulin μ-binding protein 2, which contains 993 amino acids (109,149 Da) (Maystadt et al., 2004). Its function is not fully understood. The *IGHMBP2* gene contains four domains: a DNA/RNA helicase domain, an R3H domain, a zinc finger domain, a DEXDc domain and an AN1-type zinc finger motif (Vadla et al., 2023). Among them, the DNA/RNA helicase domain contains two recA domains (Domains 1A and 2A) and at least seven highly conserved amino acid motifs (Lim et al., 2012). The synergistic effect of helicase and R3H domains leads to increased RNA binding affinity, thereby enhancing its ATPase activity (Rzepnikowska and Kochanski, 2021). Most of the missense mutations causing SMARD1 have been found in the helicase domain, indicating that this domain may play a central role in the pathogenesis of the disease (Viguier et al., 2019). Lim et al. (2012). analyzed seven missense mutations located in the ATPase domain through bioinformatics and found that they can affect ATPase activity by disrupting ATP binding/hydrolysis or by reducing the structural stability of the helicase motor. In this study, we compared the number of non-truncated mutations between SMARD1 and CMT2S located in the two RecA-like domains (Domains 1A and 2A) and found a statistically significant difference (χ^2^ = 6.893, *p* = 0.009). There were significantly more mutations in SMARD1 in the two RecA-like domains (Figure 1), which further confirms the important role of the two RecA-like domains in causing SMARD1.

Charcot-Marie-Tooth disease (CMT) is a series of polygenic syndromes that cause progressive length-dependent degeneration of peripheral sensory and/or motor fibers (Bolino and D'Antonio, 2023). The disease is associated with more than 80 different genes (Jani-Acsadi et al., 2015), including *IGHMBP2* mutations. CMT2 cases involving *IGHMBP2* mutations usually have mild myelin fiber involvement (McCray and Scherer, 2021). It is currently believed that in CMT2, mutations of the *IGHMBP2* gene are mainly a combination of nonsense mutations in the 5′ region of the gene and truncating, missense or homozygous mutations in the last exon (Saladini et al., 2020). Truncating mutations in these positions can cause CMT2S because different combinations of mutations result in different amounts of residual protein (Yuan et al., 2017). In this study, we conducted a literature review and found a statistically significant difference in the number of *IGHMBP2* truncating mutations located in the last exon between CMT2S and SMARD1 (χ^2^ = 8.971, *p* = 0.012), with significantly more mutations in CMT2S (Table 2). This indicates that the *IGHMBP2* gene truncating mutations located in the last exon are a hotspot area for mutations leading to CMT2S.

Interestingly, we compared the number of homozygous mutations between SMARD1 and CMT2S and found that whether it was a homozygous or compound heterozygous mutation of the *IGHMBP2* gene, there was no significant difference in their effect on causing SMARD1 or CMT2S, with no statistically significant differences observed (Tables 1, 2). This suggests that the heterozygosity of mutations is not a significant factor in causing SMARD1 or CMT2S. In all the homozygous variants, it was predicted that these variants caused significant 3D structural changes, leading to a decrease in protein stability. The only exception is the hemizygous variant p.Phe369Leu, which did not show any hydrogen bond changes. According to the report by Guenther et al. (2004), the p.Phe369Leu was located in the trans position of the heterozygous deletion of exons 6–13 (Guenther et al., 2004). The p.Phe369Leu mutation in the *IGHMBP2* gene is classified as “likely pathogenic” according to the ACMG guidelines, with the following evidences: PM1: The variant is located in the Helicase superfamily 1/2, ATP-binding domain; PM3: The variant forms a compound heterozygous mutation with the deletion of exons 6–13, positioned in trans; PM2_Supporting: The variant is not found in population frequency databases such as gnomAD, 1,000 Genomes, and ExAC; PP3: The variant is predicted by REVEL with a score of 0.683, falling within the range of 0.644–0.773 (Pejaver et al., 2022). Based on these ACMG criterias, it is evident that the PM3 evidence, i.e., the variant being in trans with the deletion of exons 6–13, plays a crucial role in classifying p.Phe369Leu as “likely pathogenic.” Without this evidence, the variant would be classified as “variant of uncertain significance (VUS).” Moreover, according to protein 3D structure predictions, p.Phe369Leu does not show any hydrogen bond changes, suggesting that the impact of this variant on the protein’s spatial structure is limited. Therefore, the influence of p.Phe369Leu on gene function may be minimal, and the manifestation of SMARD1-related phenotypes in the patient is more likely due to the allelic variant deletion of exons 6–13. Consequently, haploinsufficiency may also be a potential mechanism for disease caused by *IGHMBP2* defects.

Another noteworthy mutation combination is the compound heterozygous mutation of c.1060G ≥ A (p.Gly354Ser) and c.2356delG (p.Ala786ProfsTer45), which is present in two different cases (Supplementary Table S1), and leads to different types of diseases. In the case reported by Zhang et al. (2017), this compound heterozygous mutation caused SMARD1, while in the case reported by Tsang et al. (2020), it led to CMT2S. This indicates that there are differences in phenotypic expression in diseases related to the *IGHMBP2* gene in non-homozygous mutations. However, there are also differences between these two cases. In the case reported by Zhang et al. (2017), the double heterozygous mutation of the *IGHMBP2* gene is a definite compound heterozygous mutation, while in the case reported by Tsang et al. (2020), it is not clear whether the double heterozygous mutation of this gene is a compound heterozygous mutation because the c.2356delG (p.Ala786ProfsTer45) mutation has not been parentally verified, and it cannot be determined whether it is located at the trans position of the paternal c.1060G ≥ A (p.Gly354Ser) mutation. Therefore, in Tsang et al. (2020), it cannot be ruled out that this double heterozygous mutation is not a compound heterozygous mutation and that other mutations of the *IGHMBP2* gene causing CMT2S may have been missed.

In our study, homozygous truncating mutations in the *IGHMBP2* gene, except for the p.Lys868ProfsTer109 (Wagner et al., 2015), were found to cause SMARD1. Analysis of the gene structure and protein functional regions of in-trans variants in 6 hotspot *IGHMBP2* mutations showed that all truncating mutations, except for the p.Arg971GlufsTer4 (Cottenie et al., 2014), caused SMARD1 in affected individuals. The p.Lys868ProfsTer109 and p.Arg971GlufsTer4 both results in a milder form of CMT2S, possibly because their premature termination codons (PTCs) are located in residue 977 and 975, respectively, at the 3′ end of the coding sequence, allowing for the expression of a shortened functional protein and escaping nonsense-mediated mRNA decay (NMD) of the transcripts (Lindeboom et al., 2016, 2019; Hoek et al., 2019). Therefore, we hypothesize that the complete LOF mechanism of the *IGHMBP2* gene defect may be an important cause of SMARD1. However, whether LOF mutations in a single allele of the gene can cause SMARD1 is likely regulated by other complex factors.

## Conclusion

In conclusion, this study has explored the proportion of *IGHMBP2* gene variants leading to SMARD1 and CMT2S through literature search. Non-truncating variants located in two RecA-like domains (Domains 1A and 2A) of the *IGHMBP2* gene were found to be the hotspots for SMARD1, while truncating variants located in the last exon of the *IGHMBP* gene were identified as the hotspots for CMT2S. The complete LOF mechanism of the *IGHMBP2* gene defect may be an important cause of SMARD1. These findings provide further support for the diagnosis and genetic counseling of *IGHMBP2*-related diseases in clinical settings. However, the specific pathogenic mechanisms underlying these findings need to be investigated further in future studies.

## Data availability statement

The original contributions presented in the study are included in the article/Supplementary material, further inquiries can be directed to the corresponding author.

## Author contributions

YT investigated and wrote the original draft. JX revised the paper. YS organized the data. EY designed the original research. All authors contributed to the article and approved the submitted version.
