# Supplementary material for: Exploring the relationship between IGHMBP2 gene mutations and spinal muscular atrophy with respiratory distress type 1 and Charcot-Marie-Tooth disease type 2S: a systematic review
Source: Front Neurosci. 2023 Nov 17;17:1252075. doi: 10.3389/fnins.2023.1252075 (PMC10690808; doi:10.3389/fnins.2023.1252075)
Supplement: Supplementary file 1 [file Table_1.DOCX]

| Supplementary Table.1 *IGHMBP2* gene mutations in 52studies were included and classified through ACMG guidelines | | | | | | | | | | |  |
| --- | --- | --- | --- | --- | --- | --- | --- | --- | --- | --- | --- |
| No. | Study | Study types | No. of Probands | Variant(s) | Heterozygosity | Variant type(s) | ACMG Classification(s) | Whether is it in RecA-like domains(domains 1A and 2A) | Whether is it in in the last exon | Disease(s) |  |
| 1 | Serdar Pekuz,et al[PMID:35611426] | Case report | 1 | c.1738G>A (p.Val580Ile) | Hom | Missense | Pathogenic | Yes | No | SMARD1 |  |
| 2 | Michela Taiana, et al[PMID: 35086940] | Letter | 1 | c.1060G>A (p.Gly354Ser) | Het | Missense | Likely pathogenic | Yes | No | SMARD1 |  |
|  |  |  |  | c.129delC(p.Gly44AlafsTer5) | Het | Frameshift | Pathogenic | No | No |  |  |
|  |  |  | 1 | c.1915G>A (p.Ala639Thr) | Hom | Missense | Pathogenic | Yes | No | SMARD1 |  |
|  |  |  | 1 | c.121delC ( p.Gln41ArgfsTer8 ) | Het | Frameshift | Pathogenic | No | No | SMARD1 |  |
|  |  |  |  | c.439C>T (p.Arg147Ter) | Het | Nonsense | Pathogenic | No | No |  |  |
|  |  |  | 1 | c.2125C>T(p.Gln709Ter ) | Hom | Nonsense | Pathogenic | No | No | SMARD1 |  |
|  |  |  | 1 | c.653delC (p.Thr218MetfsTer15) | Het | Frameshift | Pathogenic | Yes | No | SMARD1 |  |
|  |  |  |  | c.1082T>C(p.Leu361Pro) | Het | Missense | Pathogenic | Yes | No |  |  |
|  |  |  | 1 | c.138T>A(p.Cys46Ter) | Het | Nonsense | Pathogenic | No | No | SMARD1 |  |
|  |  |  |  | c.1915G>A(p.Ala639Thr ) | Het | Missense | Pathogenic | Yes | No |  |  |
|  |  |  | 1 | c.86+1G>T | Het | Splice | Pathogenic | No | No | SMARD1 |  |
|  |  |  |  | c.298C>T(p.Gln100Ter) | Het | Nonsense | Pathogenic | No | No |  |  |
|  |  |  | 1 | c.2611+1G>T | Hom | Splice | Pathogenic | No | No | SMARD1 |  |
| 3 | M J Xiao, et al[PMID:34986626] | Case report | 1 | c.1202A>G(p.His401Arg) | Het | Missense | Likely pathogenic | Yes | No | CMT2S |  |
|  |  |  |  | c.1693G>A(p.Asp565Asn) | Het | Missense | Pathogenic | Yes | No |  |  |
| 4 | Muhammad Saeed, et al[PMID:34794294] | Case report | 1 | c.797dupG(p.His267ThrfsTer47) | Hom | Frameshift | Pathogenic | Yes | No | SMARD1 |  |
| 5 | Soumya V Chandrasekharan,et al[PMID:34668123] | Case report | 2 | c.1198G>A(p.Asp400Asn) | Hom | Missense | Pathogenic | Yes | No | CMT2S |  |
| 6 | Andre Megarbane,et al[PMID:34602496] | Retrospective study | 3 | c.1540G > A(p.Glu514Lys) | Hom | Missense | Pathogenic | Yes | No | SMARD1 |  |
|  |  |  | 2 | c.62G > T(p.Arg21Ile) | Hom | Missense | Pathogenic | No | No | CMT2S |  |
| 7 | Yongzhi Xie, et al[PMID:34255403] | Retrospective study | 1 | c.1814G>A(p.Arg605Gln ) | Het | Missense | Likely pathogenic | Yes | No | CMT2S |  |
|  |  |  |  | c.575T>A(p.Leu192Gln) | Het | Missense | Likely pathogenic | Yes | No |  |  |
|  |  |  | 1 | c.2215delA (p.Ser739AlafsTer23) | Het | Frameshift | Pathogenic | No | No | CMT2S |  |
|  |  |  |  | c.2784+1G>A | Het | Splice | Pathogenic | No | No |  |  |
|  |  |  | 1 | c.272T>C(p.Leu91Pro ) | Het | Missense | Likely pathogenic | No | No | CMT2S |  |
|  |  |  |  | c.1924T>C(p.Tyr642His) | Het | Missense | Likely pathogenic | Yes | No |  |  |
|  |  |  | 1 | c.989T>C(p.Leu330Pro) | Het | Missense | Likely pathogenic | No | No | CMT2S |  |
|  |  |  |  | c.1196G>A(p.Gly399Asp) | Het | Missense | Likely pathogenic | Yes | No |  |  |
| 8 | Kevin J Felice,et al[PMID:34232518] | Retrospective study | 1 | c.1540G > A(p.Glu514Lys) | Het | Missense | Pathogenic | Yes | No | CMT2S |  |
|  |  |  |  | c.1582G>A(p.Ala528Thr) | Het | Missense | Pathogenic | Yes | No |  |  |
| 9 | Beatrice Berti,et al[PMID:33847972] | Case report | 1 | c.1540G > A(p.Glu514Lys) | Hom | Missense | Pathogenic | Yes | No | SMARD1 |  |
| 10 | Ethan E Bodle,et al[PMID:33189025] | Case report | 1 | c.449+1G>A | Het | Splice | Pathogenic | No | No | SMARD1 |  |
|  |  |  |  | c.712-610A>G | Het | Intron | Likely pathogenic | Yes | No |  |  |
| 11 | Andrea Cortese,et al[PMID:31827005] | Retrospective study | 2 | c.1325A>G(p.Tyr442Cys) | Hom | Missense | Pathogenic | Yes | No | SMARD1 |  |
|  |  |  | 1 | c.1488C>A(p.Cys496Ter) | Het | Nonsense | Pathogenic | Yes | No | CMT2S |  |
|  |  |  |  | c.2911_2912delAG(p.Arg971GlufsTer4) | Het | Frameshift | Pathogenic | No | Yes |  |  |
|  |  |  | 1 | c.595G>C(p.Ala199Pro) | Het | Missense | Pathogenic | Yes | No | CMT2S |  |
|  |  |  |  | c.1478C>T(p.Thr493Ile) | Het | Missense | Pathogenic | Yes | No |  |  |
| 12 | Mojdeh Habibi Zoham, et al[PMID:31073488] | Case report | 1 | c.257–1G >T | Hom | Splice | Pathogenic | No | No | SMARD1 |  |
| 13 | Thomas A Cassini,et al[PMID:31020813] | Case report | 1 | c.1235+894C>A | Het | Intron | Likely pathogenic | Yes | No | CMT2S |  |
|  |  |  |  | c.1730T>C(p.Leu577Pro) | Het | Missense | Pathogenic | Yes | No |  |  |
| 14 | Young A Kim, et al[PMID:30863264] | Case report | 1 | c.1273C>T(p.Arg425Cys) | Hom | Missense | Pathogenic | Yes | No | SMARD1 |  |
| 15 | Richa Kulshrestha,et al[PMID:30409445] | Case report | 1 | c.1156T>C(p.Trp386Arg) | Het | Missense | Pathogenic | Yes | No | Atypical CMT2S |  |
|  |  |  |  | c.2747G>A(p.Cys916Tyr) | Het | Missense | Likely pathogenic | No | No |  |  |
| 16 | Pedro J Tomaselli,et al[PMID:30385095] | Case report | 1 | c.1325A>G(p.Tyr442Cys) | Hom | Missense | Pathogenic | Yes | No | SMARD1 |  |
| 17 | Annie Ting Gee Chiu,et al[PMID:29761130] | Case report | 1 | c.2362C>T(p.Arg788Ter) | Het | Nonsense | Pathogenic | No | No | SMARD1 |  |
|  |  |  |  | c.2048delG(p.Gly683AlafsTer50) | Het | Frameshift | Pathogenic | No | No |  |  |
| 18 | Shuiyan Wu,et al[PMID:29653221] | Case report | 1 | c.1737C>A(p.Phe579Leu) | Het | Missense | Pathogenic | Yes | No | Atypical SMARD1 |  |
|  |  |  |  | c.688C>G(p.Gln230Glu) | Het | Missense | Likely pathogenic | Yes | No |  |  |
| 19 | Yoshitomo Yasui,et al[PMID:29575095] | Case report | 1 | c.1537+1G>A | Het | Splice | Pathogenic | No | No | SMARD1 |  |
|  |  |  |  | c.1586G>A(p.Gly529Asp) | Het | Missense | Likely pathogenic | Yes | No |  |  |
| 20 | Maike F Dohrn[PMID:28902413] | Retrospective study | 1 | c.1478C>T(p.Thr493Ile) | Het | Missense | Pathogenic | Yes | No | CMT2S |  |
|  |  |  |  | c.547+1G>A | Het | Splice | Pathogenic | Yes | No |  |  |
|  |  |  | 1 | c.1488C>A(p.Cys496Ter) | Het | Nonsense | Pathogenic | Yes | No | CMT2S |  |
|  |  |  |  | c.1720G>A(p.Ala574Thr) | Het | Missense | Likely pathogenic | Yes | No |  |  |
|  |  |  | 1 | c.439C>T(p.Arg147Ter) | Het | Nonsense | Pathogenic | No | No | CMT2S |  |
|  |  |  |  | c.791G>A(p.Arg264His) | Het | Missense | Likely pathogenic | Yes | No |  |  |
| 21 | Biao Zhang,et al[PMID:28397221] | Case report | 1 | c.1060G>A(p.Gly354Ser) | Het | Missense | Pathogenic | Yes | No | SMARD1 |  |
|  |  |  |  | c.2356delG (p.Ala786ProfsTer45) | Het | Frameshift | Pathogenic | No | No |  |  |
| 22 | Jun-Hui Yuan[PMID:28202949] | Retrospective study | 1 | c.1034C>A(p.Ala345Glu) | Het | Missense | Likely pathogenic | No | No | CMT2S |  |
|  |  |  |  | c.1783C>T(p.Arg595Trp) | Het | Missense | Likely pathogenic | Yes | No |  |  |
|  |  |  | 1 | c.344C>T(p.Thr115Met) | Het | Missense | Pathogenic | No | No | CMT2S |  |
|  |  |  |  | c.1195G>A(p.Gly399Ser) | Het | Missense | Likely pathogenic | Yes | No |  |  |
|  |  |  |  | c.1060+5G>C | Het | Intron | Likely pathogenic | Yes | No |  |  |
|  |  |  | 1 | c.826C>T(p.Gln276Ter) | Het | Nonsense | Pathogenic | No | No | SMARD1 |  |
|  |  |  |  | c.1702C>T(p.Gln568Ter) | Het | Nonsense | Pathogenic | Yes | No |  |  |
|  |  |  | 1 | c.2759A>G(p.Tyr920Cys) | Hom | Missense | Likely pathogenic | No | No | CMT2S |  |
| 23 | Lei Liu,et al[PMID:28065684] | Retrospective study | 1 | c.1235+3A>G(p.Ala355LeufsTer10) | Hom | Frameshift | Pathogenic | Yes | No | CMT2S |  |
|  |  |  | 1 | c.1737C>A(p.Phe579Leu) | Het | Missense | Pathogenic | Yes | No | CMT2S |  |
|  |  |  |  | c.2597_2598delAG(p.Lys868SerfsTer16) | Het | Frameshift | Likely pathogenic | No | No |  |  |
|  |  |  | 1 | c.2356delG(p.Ala786ProfsTer45) | Het | Frameshift | Pathogenic | No | No | CMT2S |  |
|  |  |  |  | c.1489G>A(p.Gly497Arg) | Het | Missense | Likely pathogenic | Yes | No |  |  |
|  |  |  | 1 | c.1909C>T(p.Arg637Cys) | Het | Missense | Pathogenic | Yes | No | CMT2S |  |
|  |  |  |  | c.1061-2A>G | Het | Splice | Pathogenic | Yes | No |  |  |
| 24 | Lokesh Lingappa,et al[PMID:27570397] | Case report | 1 | c.958C>T(p.Arg320Ter) | Hom | Nonsense | Pathogenic | No | No | SMARD1 |  |
|  |  |  |  |  |  |  |  |  |  |  |  |
|  |  |  |  |  |  |  |  |  |  |  |  |
|  |  |  | 1 | c.958C>T(p.Arg320Ter) | Hom | Nonsense | Pathogenic | No | No | SMARD1 |  |
| 25 | Christeen Ramane J Pedurupillay,et al[PMID:27450922] | Case report | 1 | c.2T>C(p.Met1?) | Hom | Start Codon | Pathogenic | No | No | SMARD1 |  |
|  |  |  |  | c.861C>G(p.Ser287Arg) | Hom | Missense | Likely pathogenic | No | No |  |  |
|  |  |  | 2 | c.1478C>T(p.Thr493Ile) | Het | Missense | Pathogenic | Yes | No | CMT2S and SMARD1(also has  Kabuki syndrome) |  |
|  |  |  |  | c.983_987delAAGAA(p.Lys328ThrfsTer46) | Het | Frameshift | Pathogenic | No | No |  |  |
|  |  |  | 1 | c.449+1G>T | Hom | Splice | Pathogenic | No | No | CMT2S |  |
| 26 | Xinghua Luan,et al[PMID:26922252] | Case report | 1 | c.344C>T(p.Thr115Met) | Het | Missense | Pathogenic | No | No | CMT2S |  |
|  |  |  |  | c.1737C>A(p.Phe579Leu) | Het | Missense | Pathogenic | Yes | No |  |  |
| 27 | Beatriz San Millan[PMID:26709713] | Case report | 1 | c.439C>T(p.Arg147Ter) | Het | Nonsense | Pathogenic | No | No | SMARD1 |  |
|  |  |  |  | c.1488C>A(p.Cys496Ter) | Het | Nonsense | Pathogenic | Yes | No |  |  |
| 28 | Justin D Wagner[PMID:26298607] | Case report | 2 | c.2601_2604del(p.Lys868ProfsTer109) | Hom | Frameshift | Pathogenic | No | No | CMT2S |  |
| 29 | Mark James Hamilton[PMID:25454169] | Case report | 1 | c.1478C>T(p.Thr493Ile) | Het | Missense | Pathogenic | Yes | No | SMARD1 |  |
|  |  |  |  | c.464T>A(p.Leu155Gln) | Het | Missense | Likely pathogenic | No | No |  |  |
| 30 | Ellen Cottenie,et al[PMID:25439726] | Retrospective study | 2 | c.138T>A(p.Cys46Ter) | Het | Nonsense | Pathogenic | No | No | CMT2S |  |
|  |  |  |  | c.2911_2912delAG(p.Arg971GlufsTer4) | Het | Frameshift | Pathogenic | No | Yes |  |  |
|  |  |  | 1 | c.138T>A(p.Cys46Ter) | Het | Nonsense | Pathogenic | No | No | CMT2S |  |
|  |  |  |  | c.2911_2912delAG(p.Arg971GlufsTer4) | Het | Frameshift | Pathogenic | No | Yes |  |  |
|  |  |  | 2 | c.604T>G(p.Phe202Val) | Het | Missense | Pathogenic | Yes | No | CMT2S |  |
|  |  |  |  | c.138T>A(p.Cys46Ter) | Het | Nonsense | Pathogenic | No | No |  |  |
|  |  |  | 1 | c.1591C>A(p.Pro531Thr) | Het | Missense | Likely pathogenic | Yes | No | CMT2S |  |
|  |  |  |  | c.1738G>A(p.Val580Ile) | Het | Missense | Pathogenic | Yes | No |  |  |
|  |  |  | 1 | c.1813C>T(p.Arg605Ter) | Het | Nonsense | Pathogenic | Yes | No | CMT2S |  |
|  |  |  |  | c.2770C>T(p.His924Tyr) | Het | Missense | Likely pathogenic | No | No |  |  |
|  |  |  | 1 | c.238A>G( p.Ser80Gly) | Het | Missense | Likely pathogenic | No | No | CMT2S |  |
|  |  |  |  | c.1488C>A(p.Cys496Ter) | Het | Nonsense | Pathogenic | Yes | No |  |  |
|  |  |  | 1 | c.1156T>C(p.Trp386Arg) | Het | Missense | Pathogenic | Yes | No | CMT2S |  |
|  |  |  |  | c.2911_2912delAG(p.Arg971GlufsTer4) | Het | Frameshift | Pathogenic | No | Yes |  |  |
|  |  |  | 1 | c.2968_2980del(p.990_994del) | Hom | Inframe | Likely pathogenic | No | Yes | CMT2S |  |
|  |  |  | 2 | c.1118T>G(p.Val373Gly) | Het | Missense | Pathogenic | Yes | No | CMT2S |  |
|  |  |  |  | c.1582G>A(p.Ala528Thr) | Het | Missense | Pathogenic | Yes | No |  |  |
|  |  |  | 1 | c.1813C>T(p.Arg605Ter) | Hemi | Nonsense | Pathogenic | Yes | No | CMT2S |  |
|  |  |  |  | Deletion IGHMBP2 | Het | NA | Pathogenic | Yes | No |  |  |
| 31 | Chunxi Han,et al[PMID:25280635] | Case report | 1 | c.48delG(p.Leu17TrpfsTer21) | Het | Frameshift | Pathogenic | No | No | SMARD1 |  |
|  |  |  |  | c.1730T>G(p.Leu577Arg) | Het | Missense | Likely pathogenic | Yes | No |  |  |
| 32 | Maria Jędrzejowska,et al[PMID:24388491] | Case report | 1 | c.1615_1623del9(p.Ser539_Tyr541del) | Het | Inframe | Likely pathogenic | Yes | No | SMARD1 |  |
|  |  |  |  | c.595G>C(p.Ala199Pro) | Het | Missense | Pathogenic | Yes | No |  |  |
|  |  |  | 2 | c.439C>T(p.Arg147Ter) | Het | Nonsense | Pathogenic | No | No | SMARD1 |  |
|  |  |  |  | c.1794C>A(p.Asn598Lys) | Het | Missense | Pathogenic | Yes | No |  |  |
|  |  |  | 1 | c.1682T>C(p.Ile561Thr) | Het | Missense | Likely pathogenic | Yes | No | SMARD1 |  |
|  |  |  |  | c.1336C>T(p.Gln446Ter) | Het | Nonsense | Pathogenic | Yes | No |  |  |
| 33 | Astrid Blaschek,et al[PMID:24342282] | Case report | 1 | c.676G>T(p.Glu226Ter) | Het | Nonsense | Pathogenic | Yes | No | SMARD1 |  |
|  |  |  |  | c.2083A>T(p.Lys695Ter) | Het | Nonsense | Pathogenic | No | No |  |  |
| 34 | Xiang Lin,et al[PMID:24022109] | Retrospective study | 1 | c.1817G>A(p.Arg606His) | Het | Missense | Likely pathogenic | Yes | No | SMARD1 |  |
|  |  |  |  | c.711+1G>C | Het | Splice | Pathogenic | Yes | No |  |  |
| 35 | Aziz Majid,et al[PMID:23560007] | Case report | 1 | c.455T>C(p.Leu152Pro) | Hom | Missense | Likely pathogenic | No | No | SMARD1 |  |
| 36 | Ivan Litvinenko,et al[PMID:23449687] | Case report | 1 | c.780delG(p.Gln260HisfsTer24) | Het | Frameshift | Pathogenic | Yes | No | SMARD1 |  |
|  |  |  |  | c.1488C>A(p.Cys496Ter) | Het | Nonsense | Pathogenic | Yes | No |  |  |
| 37 | M Chalançon,et al[PMID:22981475] | Case report | 1 | c.1488C>A(p.Cys496Ter) | Het | Nonsense | Pathogenic | Yes | No | SMARD1 |  |
|  |  |  |  | c.2785-2A>G | Het | Splice | Likely pathogenic | No | Yes |  |  |
| 38 | Cyril Gitiaux,et al[PMID:22791546] | Case report | 1 | c.2611+1G>T | Het | Splice | Pathogenic | No | No | SMARD1 |  |
|  |  |  |  | c.1273C>T(p.Arg425Cys) | Het | Missense | Pathogenic | Yes | No |  |  |
| 39 | Maria Eckart,et al[PMID:22157136] | Retrospective study | 1 | c.2611+1G>T | Hom | Splice | Pathogenic | No | No | SMARD1 |  |
|  |  |  | 1 | c.1738G>A(p.Val580Ile) | Hom | Missense | Pathogenic | Yes | No | SMARD1 |  |
|  |  |  | 1 | c.1708C>T(p.Arg570Ter) | Het | Nonsense | Pathogenic | Yes | No | SMARD1 |  |
|  |  |  |  | c.1826C>A(p.Ala609Glu) | Het | Missense | Likely pathogenic | Yes | No |  |  |
|  |  |  | 1 | c.1478C>T( p.Thr493Ile) | Het | Missense | Likely pathogenic | Yes | No | SMARD1 |  |
|  |  |  |  | c.2363C>T(p.Arg788Ter) | Het | Nonsense | Pathogenic | No | No |  |  |
|  |  |  | 1 | c.638A>G(p.His213Arg) | Hom | Missense | Pathogenic | Yes | No | SMARD1 |  |
|  |  |  | 1 | c.121delC(p.Gln41ArgfsTer8) | Het | Frameshift | Pathogenic | No | No | SMARD1 |  |
|  |  |  |  | c.1060G>A(p.Gly354Ser) | Het | Missense | Pathogenic | Yes | No |  |  |
| 40 | Maria F Messina,et al[PMID:22099258] | Case report | 1 | c.2784+1G>T | Hom | Splice | Pathogenic | No | No | Atypical SMARD1 |  |
| 41 | Julie Baughn,et al[PMID:21360834] | Case report | 1 | c.1060+2T>C | Het | Splice | Pathogenic | Yes | No | SMARD1 |  |
|  |  |  |  | c.1737C>A(p.Phe579Leu) | Het | Missense | Pathogenic | Yes | No |  |  |
| 42 | Tyler Mark Pierson,et al[PMID:21353777] | Case report | 1 | c.1082T>C(p.Leu361Pro) | Het | Missense | Pathogenic | Yes | No | Atypical SMARD1 |  |
|  |  |  |  | c.1144G>A(p.Glu382Lys) | Het | Missense | Pathogenic | Yes | No |  |  |
| 43 | Abdulaziz AlSaman,et al[PMID:20197267] | Case report | 1 | c.958C>T(p.Arg320Ter) | Hom | Nonsense | Pathogenic | No | No | SMARD1 |  |
| 44 | S Joseph,et al[PMID:19157874] | Case report | 2 | c.1478C>T(p.Thr493Ile) | Het | Missense | Pathogenic | Yes | No | SMARD1 |  |
|  |  |  |  | c.1488C>A(p.Cys496Ter) | Het | Nonsense | Pathogenic | Yes | No |  |  |
| 45 | Ulf-Peter Guenther,et al[PMID:17431882] | Retrospective study | 1 | c.50T>C(p.Leu17Pro) | Het | Missense | Likely pathogenic | No | No | SMARD1 |  |
|  |  |  |  | c.1488C>A(p.Cys496Ter) | Het | Nonsense | Pathogenic | Yes | No |  |  |
|  |  |  | 1 | c.661delA(p.Thr221ProfsTer12) | Het | Frameshift | Pathogenic | Yes | No | SMARD1 |  |
|  |  |  |  | c.1415T>C(p.Leu472Pro) | Het | Missense | Likely pathogenic | Yes | No |  |  |
|  |  |  | 1 | c.1156T>C(p.Trp386Arg) | Het | Missense | Pathogenic | Yes | No | SMARD1 |  |
|  |  |  |  | c.1813C>T(p.Arg605Ter) | Het | Nonsense | Pathogenic | Yes | No |  |  |
|  |  |  | 1 | c.1235+3A>G(p.Ala355LeufsTer10) | Het | Frameshift | Pathogenic | Yes | No | SMARD1 |  |
|  |  |  |  | c.1334A>C(p.His445Pro) | Het | Missense | Likely pathogenic | Yes | No |  |  |
|  |  |  | 1 | c.1060+1G>T | Het | Splice | Pathogenic | Yes | No | SMARD1 |  |
|  |  |  |  | c.1082T>C(p.Leu361Pro) | Het | Missense | Pathogenic | Yes | No |  |  |
|  |  |  | 1 | c.1218delC(p.Thr407ProfsTer16) | Het | Frameshift | Pathogenic | Yes | No | SMARD1 |  |
|  |  |  |  | c.1708C>T(p.Arg570Ter) | Het | Nonsense | Pathogenic | Yes | No |  |  |
|  |  |  | 1 | c.616C>T(p.Gln206Ter) | Het | Nonsense | Pathogenic | Yes | No | SMARD1 |  |
|  |  |  |  | c.1877delT(p.Leu626ArgfsTer107) | Het | Frameshift | Pathogenic | Yes | No |  |  |
|  |  |  | 1 | c.163C>T(p.Gln55Ter) | Het | Nonsense | Pathogenic | No | No | SMARD1 |  |
|  |  |  |  | c.1969C>T(p.Gln657Ter) | Het | Nonsense | Pathogenic | No | No |  |  |
|  |  |  | 1 | c.904C>T(p.Gln302Ter) | Het | Nonsense | Pathogenic | No | No | SMARD1 |  |
|  |  |  |  | c.1156T>C(p.Trp386Arg) | Het | Missense | Pathogenic | Yes | No |  |  |
|  |  |  | 1 | c.388C>T(p.Arg130Ter) | Het | Nonsense | Pathogenic | No | No | SMARD1 |  |
|  |  |  |  | c.1743A>C(p.Arg581Ser) | Het | Missense | Likely pathogenic | Yes | No |  |  |
| 46 | Alberto Giannini,et al[PMID:16964485] | Case report | 1 | c.388C>T(p.Arg130Ter) | Hom | Nonsense | Pathogenic | No | No | SMARD1 |  |
| 47 | Virginia C N Wong,et al[PMID:16765827] | Case report | 1 | c.1738G>A(p.Val580Ile) | Het | Missense | Pathogenic | Yes | No | SMARD1 |  |
|  |  |  |  | c.2354_2356delG(p.Arg785_Ala786delinsThr) | Het | Inframe | Likely pathogenic | No | No |  |  |
| 48 | Ulf P Guenther,et al[PMID:15290238] | Case report | 1 | c.1107C>G(p.Phe369Leu) | Hemi | Missense | Likely pathogenic | Yes | No | SMARD1 |  |
|  |  |  |  | Deletion exon6-13 | Het | NA | Pathogenic | Yes | No |  |  |
| 49 | I Maystadt,et al[PMID:15108294] | Case report | 1 | c.647C>T(p.Pro216Leu) | Het | Missense | Likely pathogenic | Yes | No | SMARD1 |  |
|  |  |  |  | c.1807C>T(p.Arg603Cys) | Het | Missense | Likely pathogenic | Yes | No |  |  |
|  |  |  | 1 | c.587A>G(p.Gln196Arg) | Het | Missense | Likely pathogenic | Yes | No | SMARD1 |  |
|  |  |  |  | c.1909C>T(p.Arg637Cys) | Het | Missense | Pathogenic | Yes | No |  |  |
|  |  |  | 1 | c.1693G>A(p.Asp565Asn) | Het | Missense | Pathogenic | Yes | No | SMARD1 |  |
|  |  |  |  | c.2368C>T(p.Arg790Ter) | Het | Nonsense | Pathogenic | No | No |  |  |
|  |  |  | 1 | c.752T>C(p.Leu251Pro) | Het | Missense | Likely pathogenic | Yes | No | SMARD1 |  |
|  |  |  |  | c.1730T>C(p.Leu577Pro) | Het | Missense | Pathogenic | Yes | No |  |  |
| 50 | Katja Grohmann,et al[PMID:14681881] | Retrospective study | 1 | c.1488C>A(p.Cys496Ter) | Hom | Nonsense | Pathogenic | Yes | No | SMARD1 |  |
|  |  |  | 1 | c.114delA(p.Glu39SerfsTer10) | Hom | Frameshift | Pathogenic | No | No | SMARD1 |  |
|  |  |  | 1 | c.983delAAGAA(p.Glu329AsnfsTer2) | Hom | Frameshift | Pathogenic | Yes | No | SMARD1 |  |
|  |  |  | 1 | c.1540G>A(p.Glu514Lys) | Hom | Missense | Pathogenic | Yes | No | SMARD1 |  |
|  |  |  | 1 | c.575T>C(p.Leu192Pro) | Het | Missense | Likely pathogenic | Yes | No | SMARD1 |  |
|  |  |  |  | c.1277T>C((p.Leu426Pro) | Het | Missense | Likely pathogenic | Yes | No |  |  |
|  |  |  | 1 | c.388C>T(p.Arg130Ter) | Het | Nonsense | Pathogenic | No | No | SMARD1 |  |
|  |  |  |  | c.1144G>A(p.Glu382Lys) | Het | Missense | Pathogenic | Yes | No |  |  |
|  |  |  | 1 | c.1738G>A(p.Val580Ile) | Hom | Missense | Pathogenic | Yes | No | SMARD1 |  |
|  |  |  | 1 | c.1714delAA(p.K572del) | Het | Inframe | Likely pathogenic | Yes | No | SMARD1 |  |
|  |  |  |  | c.2922T>G(p.Asp974Glu) | Het | Missense | Likely pathogenic | No | Yes |  |  |
|  |  |  | 1 | c.638A>G(p.His213Arg) | Hom | Missense | Pathogenic | Yes | No | SMARD1 |  |
|  |  |  | 1 | c.1082T>C(p.Leu361Pro) | Het | Missense | Pathogenic | Yes | No | SMARD1 |  |
|  |  |  |  | c.1730T>C(p.Leu251Pro) | Het | Missense | Pathogenic | Yes | No |  |  |
|  |  |  | 1 | c.1488C>A(p.Cys496Ter) | Het | Nonsense | Pathogenic | Yes | No | SMARD1 |  |
|  |  |  |  | c.1808G>A(p.Arg603His) | Het | Missense | Likely pathogenic | Yes | No |  |  |
|  |  |  | 1 | c.1488C>A(p.Cys496Ter) | Het | Nonsense | Pathogenic | Yes | No | SMARD1 |  |
|  |  |  |  | c.1748A>T(p.Asn583Ile) | Het | Missense | Likely pathogenic | Yes | No |  |  |
|  |  |  | 1 | c.138T>A(p.Cys46Ter) | Het | Nonsense | Pathogenic | No | No | SMARD1 |  |
|  |  |  |  | c.1649insC(p.Gln550ArgfsTer9) | Het | Frameshift | Pathogenic | Yes | No |  |  |
|  |  |  | 1 | c.439C>T(p.Arg147Ter) | Het | Nonsense | Pathogenic | No | No | SMARD1 |  |
|  |  |  |  | c.1488C>A(p.Cys496Ter) | Het | Nonsense | Pathogenic | Yes | No |  |  |
|  |  |  | 1 | c.707T>G(p.Leu236Ter) | Het | Nonsense | Pathogenic | Yes | No | SMARD1 |  |
|  |  |  |  | c.1540G>A(p.Glu514Lys) | Het | Missense | Pathogenic | Yes | No |  |  |
|  |  |  | 1 | c.1488C>A(p.Cys496Ter) | Hom | Nonsense | Pathogenic | Yes | No | SMARD1 |  |
|  |  |  | 1 | c.707T>G(p.Leu236Ter) | Hom | Nonsense | Pathogenic | Yes | No | SMARD1 |  |
|  |  |  | 1 | c.707T>G(p.Leu236Ter) | Het | Nonsense | Pathogenic | Yes | No | SMARD1 |  |
|  |  |  |  | c.721T>C(p.Cys241Arg) | Het | Missense | Likely pathogenic | Yes | No |  |  |
|  |  |  | 1 | c.121delC(p.Gln41ArgfsTer8) | Het | Frameshift | Pathogenic | No | No | SMARD1 |  |
|  |  |  |  | c.388C>T(p.Arg130Ter) | Het | Nonsense | Pathogenic | No | No |  |  |
|  |  |  | 1 | c.1000G>A(p.Glu334Lys) | Hom | Missense | Likely pathogenic | Yes | No | SMARD1 |  |
|  |  |  | 1 | c.1091T>C(p.Leu364Pro) | Het | Missense | Likely pathogenic | Yes | No | SMARD1 |  |
|  |  |  |  | c.2436delT(p.Ala813ArgfsTer18) | Het | Frameshift | Pathogenic | No | No |  |  |
|  |  |  | 1 | c.2784+1G>A | Hom | Splice | Pathogenic | No | No | SMARD1 |  |
|  |  |  | 1 | c.1693G>A(p.Asp565Asn) | Het | Missense | Pathogenic | Yes | No | SMARD1 |  |
|  |  |  |  | c.1730T>C(p.Leu251Pro) | Het | Missense | Pathogenic | Yes | No |  |  |
|  |  |  | 1 | c.1756G>T(p.Gly586Cys) | Het | Missense | Likely pathogenic | Yes | No | SMARD1 |  |
|  |  |  |  | c.1909C>T(p.Arg637Cys) | Het | Missense | Pathogenic | Yes | No |  |  |
|  |  |  | 1 | c.439C>T(p.Arg147Ter) | Het | Nonsense | Pathogenic | No | No | SMARD1 |  |
|  |  |  |  | c.2362C>T(p.Arg788Ter) | Het | Nonsense | Pathogenic | No | No |  |  |
|  |  |  | 1 | c.661A>G(c.Thr221Ala) | Het | Missense | Likely pathogenic | Yes | No | SMARD1 |  |
|  |  |  |  | c.1813C>T(p.Arg605Ter) | Het | Nonsense | Pathogenic | Yes | No |  |  |
| 51 | K Grohmann,et al[PMID:11528396] | Case report | 1 | c.1540G>A(p.Glu514Lys) | Hom | Missense | Pathogenic | Yes | No | SMARD1 |  |
|  |  |  | 1 | c.638A>G(p.His213Arg) | Hom | Missense | Pathogenic | Yes | No | SMARD1 |  |
|  |  |  | 1 | c.1738G>A(p.Val580Ile) | Hom | Missense | Pathogenic | Yes | No | SMARD1 |  |
|  |  |  | 1 | c.121C>T(p.Gln41Ter) | Het | Nonsense | Pathogenic | No | No | SMARD1 |  |
|  |  |  |  | c.675delT(p.Glu226ArgfsTer7) | Het | Frameshift | Pathogenic | Yes | No |  |  |
|  |  |  | 1 | c.707T>G(p.Leu236Ter) | Hom | Nonsense | Pathogenic | Yes | No | SMARD1 |  |
|  |  |  | 1 | c.2784+1G>T | Hom | Frameshift | Pathogenic | No | No | SMARD1 |  |
| 52 | Mandy H. Y,et al[PMID:32154989] | Retrospective study | 1 | c.1060G>A(p.Gly354Ser) | Het | Missense | Pathogenic | Yes | No | CMT2S |  |
|  |  |  |  | c.2356delG (p.Ala786ProfsTer45) | Het | Frameshift | Pathogenic | No | No |  |  |
