# Supplementary material for: Exploring the relationship between IGHMBP2 gene mutations and spinal muscular atrophy with respiratory distress type 1 and Charcot-Marie-Tooth disease type 2S: a systematic review
Source: Front Neurosci. 2023 Nov 17;17:1252075. doi: 10.3389/fnins.2023.1252075 (PMC10690808; doi:10.3389/fnins.2023.1252075)
Supplement: Supplementary file 2 [file Table_2.DOCX]

| Supplementary Table.2 *IGHMBP2* gene homozygous mutations classified into SMARD1 and CMT2S | | | | | | | | | |  |
| --- | --- | --- | --- | --- | --- | --- | --- | --- | --- | --- |
| Study | Study types | No. of Probands | Variant(s) | Heterozygosity | Variant type(s) | ACMG Classification(s) | Whether is it in RecA-like domains(domains 1A and 2A) | Whether is it in in the last exon | Disease(s) |  |
| Serdar Pekuz,et al[PMID:35611426] | Case report | 1 | c.1738G>A (p.Val580Ile) | Hom | Missense | Pathogenic | Yes | No | SMARD1 |  |
|  |  | 1 | c.2125C>T(p.Gln709Ter ) | Hom | Nonsense | Pathogenic | No | No | SMARD1 |  |
|  |  | 1 | c.2611+1G>T | Hom | Splice | Pathogenic | No | No | SMARD1 |  |
| Muhammad Saeed, et al[PMID:34794294] | Case report | 1 | c.797dupG(p.His267ThrfsTer47) | Hom | Frameshift | Pathogenic | Yes | No | SMARD1 |  |
| Soumya V Chandrasekharan,et al[PMID:34668123] | Case report | 2 | c.1198G>A(p.Asp400Asn) | Hom | Missense | Pathogenic | Yes | No | CMT2S |  |
| Andre Megarbane,et al[PMID:34602496] | Retrospective study | 3 | c.1540G > A(p.Glu514Lys) | Hom | Missense | Pathogenic | Yes | No | SMARD1 |  |
|  |  | 2 | c.62G > T(p.Arg21Ile) | Hom | Missense | Pathogenic | No | No | CMT2S |  |
| Beatrice Berti,et al[PMID:33847972] | Case report | 1 | c.1540G > A(p.Glu514Lys) | Hom | Missense | Pathogenic | Yes | No | SMARD1 |  |
| Andrea Cortese,et al[PMID:31827005] | Retrospective study | 2 | c.1325A>G(p.Tyr442Cys) | Hom | Missense | Pathogenic | Yes | No | SMARD1 |  |
| Mojdeh Habibi Zoham, et al[PMID:31073488] | Case report | 1 | c.257–1G >T | Hom | Splice | Pathogenic | No | No | SMARD1 |  |
| Young A Kim, et al[PMID:30863264] | Case report | 1 | c.1273C>T(p.Arg425Cys) | Hom | Missense | Pathogenic | Yes | No | SMARD1 |  |
| Pedro J Tomaselli,et al[PMID:30385095] | Case report | 1 | c.1325A>G(p.Tyr442Cys) | Hom | Missense | Pathogenic | Yes | No | SMARD1 |  |
|  |  | 1 | c.2759A>G(p.Tyr920Cys) | Hom | Missense | Likely pathogenic | No | No | CMT2S |  |
| Lokesh Lingappa,et al[PMID:27570397] | Case report | 1 | c.958C>T(p.Arg320Ter) | Hom | Nonsense | Pathogenic | No | No | SMARD1 |  |
|  |  |  |  |  |  |  |  |  |  |  |
|  |  |  |  |  |  |  |  |  |  |  |
|  |  | 1 | c.958C>T(p.Arg320Ter) | Hom | Nonsense | Pathogenic | No | No | SMARD1 |  |
| Christeen Ramane J Pedurupillay,et al[PMID:27450922] | Case report | 1 | c.2T>C(p.Met1?) | Hom | Start Codon | Pathogenic | No | No | SMARD1 |  |
|  |  |  | c.861C>G(p.Ser287Arg) | Hom | Missense | Likely pathogenic | No | No |  |  |
|  |  | 1 | c.449+1G>T | Hom | Splice | Pathogenic | No | No | CMT2S |  |
| Justin D Wagner[PMID:26298607] | Case report | 2 | c.2601_2604del(p.Lys868ProfsTer109) | Hom | Frameshift | Pathogenic | No | No | CMT2S |  |
|  |  | 1 | c.2968_2980del(p.990_994del) | Hom | Inframe | Likely pathogenic | No | Yes | CMT2S |  |
|  |  | 1 | c.1813C>T(p.Arg605Ter) | Hemi | Nonsense | Pathogenic | Yes | No | CMT2S |  |
| Aziz Majid,et al[PMID:23560007] | Case report | 1 | c.455T>C(p.Leu152Pro) | Hom | Missense | Likely pathogenic | No | No | SMARD1 |  |
| Maria Eckart,et al[PMID:22157136] | Retrospective study | 1 | c.2611+1G>T | Hom | Splice | Pathogenic | No | No | SMARD1 |  |
|  |  | 1 | c.1738G>A(p.Val580Ile) | Hom | Missense | Pathogenic | Yes | No | SMARD1 |  |
|  |  | 1 | c.638A>G(p.His213Arg) | Hom | Missense | Pathogenic | Yes | No | SMARD1 |  |
| Maria F Messina,et al[PMID:22099258] | Case report | 1 | c.2784+1G>T | Hom | Splice | Pathogenic | No | No | Atypical SMARD1 |  |
| Abdulaziz AlSaman,et al[PMID:20197267] | Case report | 1 | c.958C>T(p.Arg320Ter) | Hom | Nonsense | Pathogenic | No | No | SMARD1 |  |
| Alberto Giannini,et al[PMID:16964485] | Case report | 1 | c.388C>T(p.Arg130Ter) | Hom | Nonsense | Pathogenic | No | No | SMARD1 |  |
| Ulf P Guenther,et al[PMID:15290238] | Case report | 1 | c.1107C>G(p.Phe369Leu) | Hemi | Missense | Likely pathogenic | Yes | No | SMARD1 |  |
| Katja Grohmann,et al[PMID:14681881] | Retrospective study | 1 | c.1488C>A(p.Cys496Ter) | Hom | Nonsense | Pathogenic | Yes | No | SMARD1 |  |
|  |  | 1 | c.114delA(p.Glu39SerfsTer10) | Hom | Frameshift | Pathogenic | No | No | SMARD1 |  |
|  |  | 1 | c.983delAAGAA(p.Glu329AsnfsTer2) | Hom | Frameshift | Pathogenic | Yes | No | SMARD1 |  |
|  |  | 1 | c.1540G>A(p.Glu514Lys) | Hom | Missense | Pathogenic | Yes | No | SMARD1 |  |
|  |  | 1 | c.1738G>A(p.Val580Ile) | Hom | Missense | Pathogenic | Yes | No | SMARD1 |  |
|  |  | 1 | c.638A>G(p.His213Arg) | Hom | Missense | Pathogenic | Yes | No | SMARD1 |  |
|  |  | 1 | c.1488C>A(p.Cys496Ter) | Hom | Nonsense | Pathogenic | Yes | No | SMARD1 |  |
|  |  | 1 | c.707T>G(p.Leu236Ter) | Hom | Nonsense | Pathogenic | Yes | No | SMARD1 |  |
|  |  | 1 | c.1000G>A(p.Glu334Lys) | Hom | Missense | Likely pathogenic | Yes | No | SMARD1 |  |
|  |  | 1 | c.2784+1G>A | Hom | Splice | Pathogenic | No | No | SMARD1 |  |
| K Grohmann,et al[PMID:11528396] | Case report | 1 | c.1540G>A(p.Glu514Lys) | Hom | Missense | Pathogenic | Yes | No | SMARD1 |  |
|  |  | 1 | c.638A>G(p.His213Arg) | Hom | Missense | Pathogenic | Yes | No | SMARD1 |  |
|  |  | 1 | c.1738G>A(p.Val580Ile) | Hom | Missense | Pathogenic | Yes | No | SMARD1 |  |
|  |  | 1 | c.707T>G(p.Leu236Ter) | Hom | Nonsense | Pathogenic | Yes | No | SMARD1 |  |
|  |  | 1 | c.2784+1G>T | Hom | Splice | Pathogenic | No | No | SMARD1 |  |
