# Supplementary material for: Exploring the relationship between IGHMBP2 gene mutations and spinal muscular atrophy with respiratory distress type 1 and Charcot-Marie-Tooth disease type 2S: a systematic review
Source: Front Neurosci. 2023 Nov 17;17:1252075. doi: 10.3389/fnins.2023.1252075 (PMC10690808; doi:10.3389/fnins.2023.1252075)
Supplement: Supplementary file 3 [file Table_3.DOCX]

Supplementary Table.3 Data summary of high frequency variants of *IGHMBP2* gene

| Variant(s) | Study | Variant(s) in trans | Disease type |
| --- | --- | --- | --- |
| c.1488C>A(p.Cys496Ter) | Andrea Cortese,et al[PMID:31827005] | c.2911_2912delAG(p.Arg971GlufsTer4) | CMT2S |
|  | Maike F Dohrn[PMID:28902413] | c.1720G>A(p.Ala574Thr) | CMT2S |
|  | Beatriz San Millan[PMID:26709713] | c.439C>T(p.Arg147Ter) | SMARD1 |
|  | Ellen Cottenie,et al[PMID:25439726] | c.238A>G(p.Ser80Gly) | CMT2S |
|  | Ivan Litvinenko,et al[PMID:23449687] | c.780delG(p.Gln260HisfsTer24) | SMARD1 |
|  | M Chalançon,et al[PMID:22981475] | c.2785-2A>G | SMARD1 |
|  | S Joseph,et al[PMID:19157874] | c.1478C>T(p.Thr493Ile) | SMARD1 |
|  | Ulf-Peter Guenther,et al[PMID:17431882] | c.50T>C(p.Leu17Pro) | SMARD1 |
|  | Katja Grohmann,et al[PMID:14681881] | c.1488C>A(p.Cys496Ter)(Hom) | SMARD1 |
|  | Katja Grohmann,et al[PMID:14681881] | c.1808G>A(p.Arg603His) | SMARD1 |
|  | Katja Grohmann,et al[PMID:14681881] | c.1478C>T(p.Thr493Ile) | SMARD1 |
|  | Katja Grohmann,et al[PMID:14681881] | c.439C>T(p.Arg147Ter) | SMARD1 |
|  | Katja Grohmann,et al[PMID:14681881] | c.1488C>A(p.Cys496Ter)(Hom) | SMARD1 |
| c.138T>A(p.Cys46Ter) | Michela Taiana, et al[PMID: 35086940] | c.1915G>A(p.Ala639Thr ) | SMARD1 |
|  | Ellen Cottenie,et al[PMID:25439726] | c.2911_2912delAG(p.Arg971GlufsTer4) | CMT2S |
|  | Ellen Cottenie,et al[PMID:25439726] | c.2911_2912delAG(p.Arg971GlufsTer4) | CMT2S |
|  | Ellen Cottenie,et al[PMID:25439726] | c.604T>G(p.Phe202Val) | CMT2S |
|  | Katja Grohmann,et al[PMID:14681881] | c.1649insC(p.Gln550ArgfsTer9) | SMARD1 |
| c.1478C>T(p.Thr493Ile) | Andrea Cortese,et al[PMID:31827005] | c.595G>C(p.Ala199Pro) | CMT2S |
|  | Maike F Dohrn[PMID:28902413] | c.547+1G>A | CMT2S |
|  | Christeen Ramane J Pedurupillay,et al[PMID:27450922] | c.983_987delAAGAA(p.Lys328ThrfsTer46) | CMT2S and SMARD1(also has  Kabuki syndrome) |
|  | Mark James Hamilton[PMID:25454169] | c.464T>A(p.Leu155Gln) | SMARD1 |
|  | Maria Eckart,et al[PMID:22157136] | c.2363C>T(p.Arg788Ter) | SMARD1 |
|  | S Joseph,et al[PMID:19157874] | c.1488C>A(p.Cys496Ter) | SMARD1 |
| c.1738G>A(p.Val580Ile) | Serdar Pekuz,et al[PMID:35611426] | c.1738G>A(p.Val580Ile)(Hom) | SMARD1 |
|  | Ellen Cottenie,et al[PMID:25439726] | c.1591C>A(p.Pro531Thr) | CMT2S |
|  | Maria Eckart,et al[PMID:22157136] | c.1738G>A(p.Val580Ile)(Hom) | SMARD1 |
|  | Virginia C N Wong,et al[PMID:16765827] | c.2354_2356delG(p.Arg785_Ala786delinsThr) | SMARD1 |
|  | Katja Grohmann,et al[PMID:14681881] | c.1738G>A(p.Val580Ile)(Hom) | SMARD1 |
|  | K Grohmann,et al[PMID:11528396] | c.1738G>A(p.Val580Ile)(Hom) | SMARD1 |
| c.439C>T(p.Arg147Ter) | Michela Taiana, et al[PMID: 35086940] | c.121delC ( p.Gln41ArgfsTer8 ) | SMARD1 |
|  | Maike F Dohrn[PMID:28902413] | c.791G>A(p.Arg264His) | CMT2S |
|  | Beatriz San Millan[PMID:26709713] | c.1488C>A(p.Cys496Ter) | SMARD1 |
|  | Maria Jędrzejowska,et al[PMID:24388491] | c.1794C>A(p.Asn598Lys) | SMARD1 |
|  | Katja Grohmann,et al[PMID:14681881] | c.1488C>A(p.Cys496Ter) | SMARD1 |
|  | Katja Grohmann,et al[PMID:14681881] | c.2362C>T(p.Arg788Ter) | SMARD1 |
| c.1540G > A(p.Glu514Lys) | Andre Megarbane,et al[PMID:34602496] | c.1540G > A(p.Glu514Lys)(Hom) | SMARD1 |
|  | Kevin J Felice,et al[PMID:34232518] | c.1582G>A(p.Ala528Thr) | CMT2S |
|  | Beatrice Berti,et al[PMID:33847972] | c.1540G > A(p.Glu514Lys)(Hom) | SMARD1 |
|  | Katja Grohmann,et al[PMID:14681881] | c.1540G > A(p.Glu514Lys)(Hom) | SMARD1 |
|  | Katja Grohmann,et al[PMID:14681881] | c.707T>G(p.Leu236Ter) | SMARD1 |
|  | K Grohmann,et al[PMID:11528396] | c.1540G > A(p.Glu514Lys)(Hom) | SMARD1 |
